# Biological treatment of biowaste as an innovative source of CO—The role of composting process

**DOI:** 10.3389/fbioe.2023.1126737

**Published:** 2023-02-09

**Authors:** Karolina Sobieraj, Sylwia Stegenta-Dąbrowska, Gang Luo, Jacek A. Koziel, Andrzej Białowiec

**Affiliations:** ^1^ Department of Applied Bioeconomy, Wrocław University of Environmental and Life Sciences, Wrocław, Poland; ^2^ Shanghai Key Laboratory of Atmospheric Particle Pollution and Prevention (LAP3), Department of Environmental Science and Engineering, Fudan University, Shanghai, China; ^3^ Shanghai Technical Service Platform for Pollution Control and Resource Utilization of Organic Wastes, Shanghai, China; ^4^ Shanghai Institute of Pollution Control and Ecological Security, Shanghai, China; ^5^ USDA-ARS Conservation and Production Research Laboratory, Bushland, TX, United States; ^6^ Department of Agricultural and Biosystems Engineering, Iowa State University, Ames, IA, United States

**Keywords:** carbon monoxide, anaerobic, aerobic, CODH, composting, bibliometric analysis

## Abstract

Carbon monoxide (CO) is an essential “building block” for producing everyday chemicals on industrial scale. Carbon monoxide can also be generated though a lesser-known and sometimes forgotten biorenewable pathways that could be explored to advance biobased production from large and more sustainable sources such as bio-waste treatment. Organic matter decomposition can generate carbon monoxide both under aerobic and anaerobic conditions. While anaerobic carbon monoxide generation is relatively well understood, the aerobic is not. Yet many industrial-scale bioprocesses involve both conditions. This review summarizes the necessary basic biochemistry knowledge needed for realization of initial steps towards biobased carbon monoxide production. We analyzed for the first time, the complex information about carbon monoxide production during aerobic, anaerobic bio-waste treatment and storage, carbon monoxide-metabolizing microorganisms, pathways, and enzymes with bibliometric analysis of trends. The future directions recognizing limitations of combined composting and carbon monoxide production have been discussed in greater detail.

## 1 Introduction

The global consumption and demand for resource-intensive goods, energy and raw materials continues to grow, largely exceeding current renewable pathways. There is a need to search for innovative and more sustainable ways to address these pressing challenges. The circular economy is now considered not as an option, but a necessity. Sustainable resource management and recovery, including waste and biomass, scaling up biotechnological and microbiological processes to biorefineries, can improve cycling loops in bioeconomy-driven future.

Current advances in circularity and bioeconomy models require continuous ground-truthing and refinement on scales that are relevant to be impactful. The scale of environmental challenges requires well-informed decisions, which must nevertheless be based on basic and applied research. Prioritizing research can enable more sustainable technologies in different sectors of the economy.

A fresh look at commonly known materials, substrates, by-products can provide “waste,” “bio-waste,” or “pollutant” a new meaning. The substrate that deserves a refreshed focus is carbon monoxide (CO). Recognized as a primary air pollutant, CO is also purposefully generated through thermochemical reactions and appreciated for its numerous industrial applications, including in metallurgy, as a component of synthesis gas, and production of common chemicals such as ethanol, methanol, hydrocarbons and aromatic compounds (Perondi et al., 2017; Yang et al., 2017).

However, it is less-known that CO can be produced biologically, as a by-product of biological waste treatment processes (Stegenta et al., 2018; Stegenta et al., 2019a; Stegenta-Dąbrowska et al., 2019). Biological processes of organic matter (OM) decomposition, both under aerobic and anaerobic conditions can generate CO. While anaerobic CO generation is relatively well understood (Oelgeschläger and Rother, 2008; Andreides et al., 2022), the aerobic is not. Yet many industrial-scale bioprocesses involve both conditions and can be difficult to control.

It has been reported that CO is present during biowaste composting in aerobic piles and bioreactors, with concentration exceeding 1,000 ppm (Stegenta et al., 2019a; Stegenta-Dąbrowska et al., 2022). Thus, there appears to be sufficient amount of CO generated in a common bio-waste treatment process that could be further explored as a pathway for industrial scale biorenewable CO production. This is particularly interesting considering that its biological production is mainly associated with the presence of microorganisms that produce the enzyme CO dehydrogenase (CODH), responsible for both the production and metabolism of CO under anaerobic conditions (Abubackar et al., 2011; Jeong et al., 2015).

Considering that in the composted pile there are both aerobic and anaerobic areas, inhabited by microorganisms capable of functioning in both conditions, important research direction-type questions arise:

(i) Is the CO production during the aerobic biowaste composting processes based on the activity of the same microorganisms and the CODH enzyme produced by them as in the case of anaerobic processes?
(ii) Can the existing knowledge gained while analyzing CO generation under anaerobic conditions, be applied for composting that is both aerobic and anaerobic?
(iii) Can a “lowly” composting become a leading process for the biobased production of valuable CO?

This review summarizes the necessary basic biochemistry knowledge needed for realization of initial steps towards biobased CO production. The limited information about CO sources, mechanisms, microorganisms involved, and optimal conditions for its formation during bio-waste aerobic biostabilization, including composting, is summarized. We analyzed for the first time, the complex information about CO production during aerobic, anaerobic bio-waste treatment and storage, CO-metabolizing microorganisms, pathways, and enzymes with bibliometric analysis of trends. The future directions recognizing limitations of combined composting and CO production have been discussed in greater detail.

## 2 Methods

The Web of Science Core Collection was searched to find journal articles (without a specific date range). “Topic,” which included title, abstract, author keywords, and Keywords Plus has been set as the search parameter. The bibliometric analysis was performed for the combination of keywords:

(i) “Carbon monoxide” + “anaerobic”,
(ii) “Carbon monoxide” + “pathway”,
(iii) “Carbon monoxide” + “CODH”,
(iv) “Carbon monoxide” + “microorganisms”,
(v) “Composting” + “carbon monoxide”,
(vi) “Composting” + “CODH”.

The data were transferred to Microsoft Excel 2007. Google Scholar was used to document the original and foundational research on this topic, which was relatively old and outside the time range of the Web of Science Core Collection (Haddaway et al., 2015). Inclusion on these older references was crucial to connect the key established facts present only in the earlier literature, bridge the gap in knowledge, and attempt to chart the future research direction.

## 3 Results

### 3.1 Bibliographic record on carbon monoxide

**FIGURE 1:**
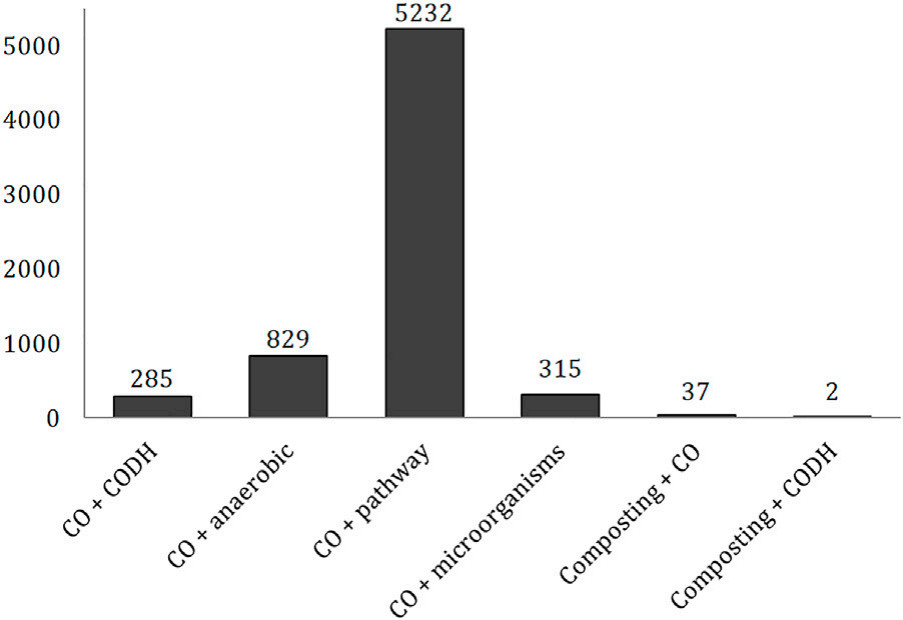
Number of publications in the Web of Science Core Collection for chosen combination of keywords.

The highest number of records was found for “carbon monoxide” + “pathway” (5,232), following by “carbon monoxide” + “anaerobic” (829; Figure 1). A much lower and similar number of scientific papers was found for the combination of “carbon monoxide” with “microorganisms” and “CODH” keywords (315 and 285, respectively). The main publishing form was research article (85%, 84%, 77%, 91%, respectively), followed by reviews (12%, 13%, 17%, 6%, respectively).

First reported studies were noted for “carbon monoxide” + “anaerobic” keywords (1955, Figure 2). The most frequently discussed topic was the CO metabolic pathways where the earliest works dated back to the 1960s and 1970s. This was followed by first publications in the field of microorganisms involved in the CO cycle (1972–1974). Two decades later, the role of the CODH enzyme started to emerge in reports from the 1991 initial studies. The key breakthroughs for each of the above-mentioned topics occurred in the 1990s and the 1st decade of the 20th century. This was evidenced by the increase of number of the articles published (by ∼870% between 1990 and 1991 for “pathway” articles, 120% in case of “CODH” keywords in 1995–96 and 100% for “microorganisms” between 2010/2011). The highest variations in the published output from year-to-year were observed for the CODH enzyme, likely due to the relatively narrow focus of this field of study.

**FIGURE 2:**
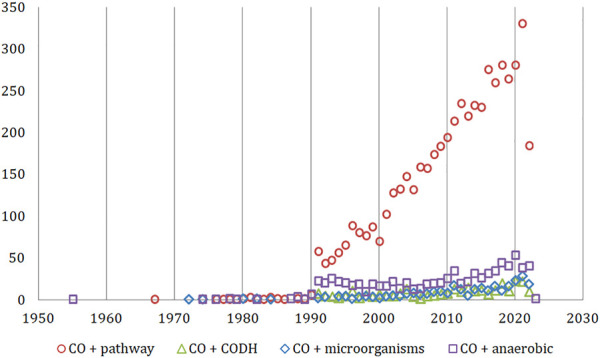
The timeline of research areas for “carbon monoxide” articles relevant to the scope of this review (Web of Science Core Collection).

### 3.2 Bibliographic record on composting and CO

While a high number of records were found for articles focusing on the CO biochemistry, the subject of composting in combination with CO and the CODH enzyme was much less discussed by researchers (Figure 1). The CO production was described in 37 articles, while the production of CODH by microorganisms in the composted biomass was reported in only two.

**FIGURE 3:**
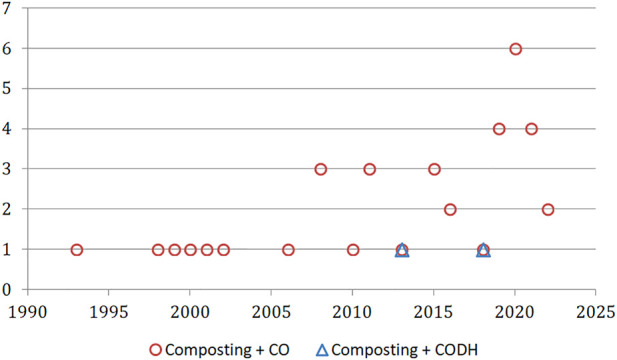
The timeline of published research for “composting” articles related to CO and CODH (Web of Science Core Collection).

The formation of CO during the aerobic waste treatment was first published in the 1990s; only a handful of studies were published until 2006, after which the number of reports on this topic increased to 3 research articles per year (Figure 3). The most popular combination of the words “composting” + “carbon monoxide” was achieved recently (up to 6 articles per year published between 2019–2022). On the other hand, after the publication of two articles on the CODH enzyme in the context of the composting process (dated 2013 and 2018), this topic was not raised again.

In general, it is important to recognize that this field of study and the scope of this review, have been accelerating rather slowly, and that large opportunities for connecting the facts and charting new research directions aiming at biobased production of CO still exist.

## 4 Discussion

### 4.1 Microorganisms involved in the CO metabolism

#### 4.1.1 Aerobic bacteria

Aerobic bacteria utilizing CO for growth were isolated from garden soil and described in (Beijerinck and van Delden, 1903) for the first time in 1903. Then, bacteria which could grow in air enriched with CO (Lantzsch, 1922), and Hydrogenomonas carboxidovorans bacteria, capable of oxidizing CO and H_2_ were found in 1953 (Kistner, 1953). CO-oxidizing bacteria were also isolated from urban soils during their enrichment with a mixture of 20% O_2_ and 80% CO in 1973 (Nozhevnikova and Zavarzin, 1973). By the end of the 20th century, additional CO oxidants were discovered, called carboxydotroph, included Actinobacteria, Proteobacteria, and Firmicutes (Gadkari et al., 1990). The growth of these bacterias was observed with increased CO concentration (>10%) (Gadkari et al., 1990). Today, carboxydotrophs are viewed as a group of bacteria that use carbon and CO energy as their sole source when present in concentrations >1% (King, 2003). Although many of their groups are not closely related, they share the same metabolic profile, based on the possibility of aerobic growth with CO levels as high as 90% (v/v) (Meyer et al., 1986). This process involves directing electrons to a CO-insensitive terminal oxidase with a high affinity for O_2_ through a CO-insensitive branch of the respiratory chain (Cypionka and Meyer, 1983).

Subsequent discoveries proved that some of the CO oxidants could not function under such extreme conditions. Due to their growth only at low CO concentration and taking up gas as an additional source of energy for survival, they were referred to as carboxidovores [including *Mesorhizobium plurifarium* and *Bradyrhizobium* spp., CPP from the rhizosphere, *Stappia aggregata* from marine sediment, *Silicibacter pomeroyi* from seawater, *Burkholderia xenovorans* from soil or *Mycobacterium* spp. RIM from volcanic soil (Weber and King, 2007)]. However, they play an important role in the biogeochemistry of CO, representing a group of facultative lithotrophs and taking CO from many natural systems, such as sediments, plant roots, or oxic soils (Moran et al., 2004).

Continued analysis of various environments, such as compost, sewage, sewage sludge, or freshwater sediment, allowed, however, to find also more carboxydotrophs, the diversity of which turned out to be very rich [incl. *Oligotropha carboxidovorans*, *Pseudomonas thermocarboxydovorans*, *Pseudomonas carboxydohydrogena*, *Bacillus schlegelii* (Krüger and Meyer, 1984)]. Most carboxydotrophs are mesophilic, with few exceptions as *P. thermocarboxydovorans* (Lyons et al., 1984), *B. schlegelii* (Krüger and Meyer, 1984), and *Streptomyces thermoautotrophicus* (Gadkari et al., 1990). However, no extreme species such as acidophiles, psychrophiles, hyperthermophiles, and extreme halophiles were reported (King and Weber, 2007). Carboxydotrophs also include plant pathogens and symbiotes (Tiquia-Arashiro, 2014).

#### 4.1.2 Anaerobic bacteria

The anaerobic carboxydotrophic bacteria that metabolize CO as the only carbon source include acetogens, methanogens, sulfate and elemental sulfur reducers, phototrophic bacteria, and hydrogenogens (Techtmann et al., 2009). Like aerobes, they are widespread in natural habitats, but their rich cultures’ preferred locations are not fully understood (Nguyen et al., 2013).

Among the groups mentioned above, researchers are most interested in the obligatory anaerobes—acetogens. Their use can lead to obtaining useful substances, such as chemicals and fuels (Henstra et al., 2007). During syngas fermentation, acetogens produce, among others, acetate, butanol, and butyrate (Jeong et al., 2015). On the other hand, ethanol from CO can generate such homoacetogens as *Alkalibaculumbacchi, Butyribacterium methylotrophicum, Clostridium ljungdahlii, Clostridium carboxidivorans* P7^T^, *Clostridium ragsdalei, C. autoethanogenum* and *Clostridium drakei* (Liu et al., 2012; Mohammadi et al., 2012). Members of acetogens have been isolated from such media as composts, wastewater, and the rhizosphere, volcanic soil, hydrotherms, water sediments, or coal heaps (King and Weber, 2007). The utilization of CO for acetogens is a way to provide energy, cellular material, but also CO_2_ and acetate (Mörsdorf et al., 1992). For example, some acetogens may consume CO as an electron donor and produce H_2_ when oxidizing this compound, such as *C. thermoaceticum* (Kerby and Zeikus, 1983). Other acetogens, including *Acetogeniumkivui*, cannot do so and only utilize CO in the material and acetate produced (Daniel et al., 1990). Acetogenic bacteria can grow at a high CO environment. The “record holders” are representatives of *Peptostreptococcus productus* (Ma et al., 1991), which can thrive at up to 90% CO (v/v), and they also show the fastest growth on CO (Mörsdorf et al., 1992).

Methanogenic bacteria are obligate anaerobes that produce CH_4_ from CO_2_, other carbon compounds, or acetate (Mörsdorf et al., 1992). This group’s first representative to utilize CO for growth was discovered in 1977 (Daniels et al., 1977). Like acetogens, methanogens use CO as a source of energy, cellular material, and CO_2_, but their main product is CH_4_ instead of acetate. Methanogens are also more sensitive to CO (Mörsdorf et al., 1992). Representatives of this group include: *Methanothermobacter thermautotrophicus, Methanosarcina barkeri*, and *M. acetivorans* (Rother et al., 2004).

The use of CO as an energy source by converting it into CO_2_ and H_2_ is characteristic of desulfuricans (Sipma et al., 2006). They are obligate anaerobes capable of autotrophic growth (Fauque et al., 1991). Their ability to oxidize CO was observed in the 1950s by Yagi’s research team (Yagi, 1959). The generated H_2_ is later used to reduce sulfates (Rabus et al., 2013). Some of these bacteria, such as *Desulfotomaculum thermobenzoicum* and *Desulfotomaculum kuznetsovii*, are also produced by acetate (Parshina et al., 2005a). Because sulfate-reducing bacteria can generally only tolerate low CO concentrations [up to a few percent (Jansen et al., 2004)], it is believed that the H_2_ production by CO oxidation (biologically-induced water gas-shift—BWGS reaction) is by detoxification (Oelgeschläger and Rother, 2008). Higher CO concentrations inhibit the growth of, among others, *Desulfotomaculum* species or *Desulfovibrio vulgaris* strain Madison (Lupton et al., 1984; Klemps et al., 1985). On the other hand, *Desulfotomaculum carboxydivorans* is capable of growth at 100% CO, which consequently draws attention to use this strain in BWGS reaction. Without sulfate, CO is converted into H_2_ and CO_2_, while in the presence of sulfate, some of the produced H_2_ is used for sulfate reduction (Parshina et al., 2005b). Thermophilic bacteria have been discovered amongst desulfuricants, including *D. thermoacetoxidans* and *T. yellowstonii, D. kuznetsovii,* and *D. thermobenzoicum* subsp. *Thermosyntrophicum* (Parshina et al., 2005a).

CO tolerance by phototrophic bacteria was noted in 1968 (Hirsch, 1968), and less than a decade later, it was discovered that CO could be the only source of carbon and energy under dark conditions for *Rubrivivaxgelatinosus* and *Rhodospirillum rubrum* (Uffen, 1976; Dashekvicz and Uffen, 1979; Uffen, 1981). It has been reported that the former is capable of developing at 100% CO in the gas-phase, and by oxidizing it, it produces CO_2_ and H_2_ (Techtmann et al., 2009). The microbial capabilities of the BWSG reaction were confirmed by (Younesi et al., 2008). *R. rubrum* showed a higher rate of CO conversion yield compared to other similar microorganisms (Alfano and Cavazza, 2018). For this reason, CO has become the subject of research on the production of biohydrogen from syngas (Kerby et al., 1995), which indicated that it requires an additional carbon source for CO conversion and growth, and it works most efficiently using acetate as a substrate (Najafpour and Younesi, 2007).

Laboratory studies of BWGS reaction conducted with *R. rubrum* were extended to industrial scale; bacteria proved to be suitable for large-scale biohydrogen production in continuous bioreactors, opening the door to the development of H_2_ production technology using living microorganisms (Alfano and Cavazza, 2018). *Citrobacter* sp. Y19 obtained three times higher level of produced H_2_ compared with *R. Rubrum* (Jung et al., 2002).

The hydrogenogens term originates in the 21st century, refers to anaerobic thermophilic bacteria and archaea that, as they grow, oxidize CO using H_2_O as an electron acceptor, producing molecular hydrogen and CO_2_ (Oelgeschläger and Rother, 2008). These reactions resemble BWGS (Oelgeschläger and Rother, 2008), and researchers suggest that high temperature facilitates hydrogenogens’ CO metabolism due to increased gas diffusion rate (Diender et al., 2015). Among the hydrogenogens, the *Carboxydothermus hydrogenoformans*, *Thermosinus carboxydivorans* or *Thermococcus* AM4 are well-known (Techtmann et al., 2009). Hydrogenogens can be found in hydrothermal, geothermal, and volcanic environments (Wu et al., 2005). *C. hydrogenoformans* have become of interest as this microorganism is likely to enable the production of biohydrogen from syngas due to the rapid growth and CO as a source of sole carbon and energy catalyzing in the dark BWGS reaction (Wu et al., 2005). Although researchers have not yet documented the ability to convert CO contained in it to H_2_, there are sources relating to pure CO use by these bacteria (Tiquia-Arashiro, 2014).

However, what is important when discussing the anaerobic conversion of CO by bacteria is the diversity and variability of microbial communities during this process, explored for syngas biomethanation. CO can be converted directly or indirectly *via* other pathways, also leading to intermediate products such as H_2_, CO_2_, formate, acetate, butanol, ethanol, propionate or butyrate (Sancho Navarro et al., 2016; Aryal et al., 2021). It is these intermediary metabolites that contribute to the development of a variety of bacterial strains in the bioreactor. However, what needs to be emphasized is that the biological reactions of CO conversion conducted by different microbial groups have different energy balances (Asimakopoulos et al., 2020). The standard change of Gibbs free energy for these biocatalytic reactions indicates that the activity of carboxydotrophic methanogens, converting CO to CO_2_ and CH_4_ is the most favorable, since the ΔGº reaches a value of −210.9 kJ·mol^-1^ [compared to −165.4 and −135.6 kJ·mol^-1^ for the next two most preferred bacteria, acetogens, and hydrogenotrophic methanogens (Grimalt-Alemany et al., 2018)]. The multiplicity of syntrophically coexisting bacteria can also be explained by the fact that they use CO both as a carbon and energy source (Asimakopoulos et al., 2020). It was also proven that bacterial CO-converting community composition changes depending on the type of substrate used in the process of syngas upgrade to biomethane; these observations were made with manure and sludge-based inoculum (Grimalt-Alemany et al., 2018).

### 4.2 CO microbiological consumption—Pathways and enzymes

The microbiological CO consumption depends on the O_2_ availability and follows the first-order kinetics (Conrad and Seiler, 1980). This was also confirmed by another report (Rich and King, 1999), where the CO consumption in anaerobic conditions was lower than that carried out in aerobic conditions. It has also been found that CO is metabolized under all oxidation-reduction conditions. All these observations are in line with our previous study, where we showed that maximum CO concentration was observed at ∼5% O_2_ in the composting pile (Stegenta-Dąbrowska et al., 2019).

However, the enzymes used under aerobic and anaerobic conditions differ in terms of chemical structure and presence of Ni-Fe clusters in active centers. CO is metabolized both under aerobic and anaerobic conditions by (Jeong et al., 2015):

(i) CO dehydrogenase (CODH)—acceptor oxidoreductase as the systematic name for the activity that catalyzes CO oxidation to CO_2_ or its reverse,
(ii) Acetyl-CoA synthase (ACS)—enzyme that assembles acetyl-CoA from enzyme-bound methyl, CO, and CoA groups,
(iii) Bifunctional (CO dehydrogenase/acetyl-CoA synthase or CODH/ACS)—neither ACS nor CODH alone would suffice because they describe only the partial reactions. CODH/ACS is preferable to ACS/CODH, because CODH precedes ACS in function.

**FIGURE 4:**
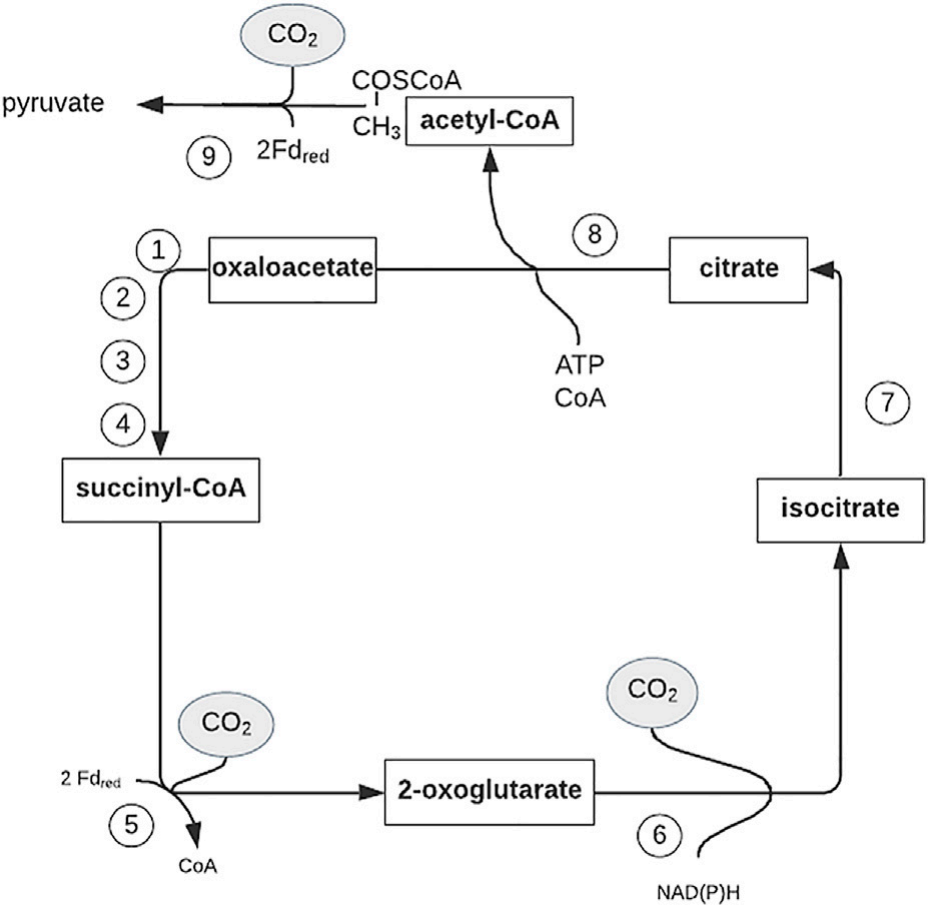
Outline of the reductive citric acid cycle for autotrophic CO_2_ fixation. Figure redrawn after reference (Hügler et al., 2005). Bold arrows indicate the reactions catalyzed by key enzymes. Enzyme activities: 1, malate dehydrogenase (EC 1.1.1.37); 2, fumarate hydratase (fumarase) (EC 4.2.1.2); 3, fumarate reductase; 4, succinyl-CoA synthetase (EC 6.2.1.5); 5, 2-oxoglutarate:ferredoxinoxidoreductase (EC 1.2.7.3); 6, isocitrate dehydrogenase (EC 1.1.1.42); 7, aconitate hydratase (aconitase) (EC 4.2.1.3); 8, ATP citrate lyase (EC 2.3.3.8); and 9, pyruvate:ferredoxin oxidoreductase (EC 1.2.7.1); Fd red, reduced ferredoxin.

**FIGURE 5:**
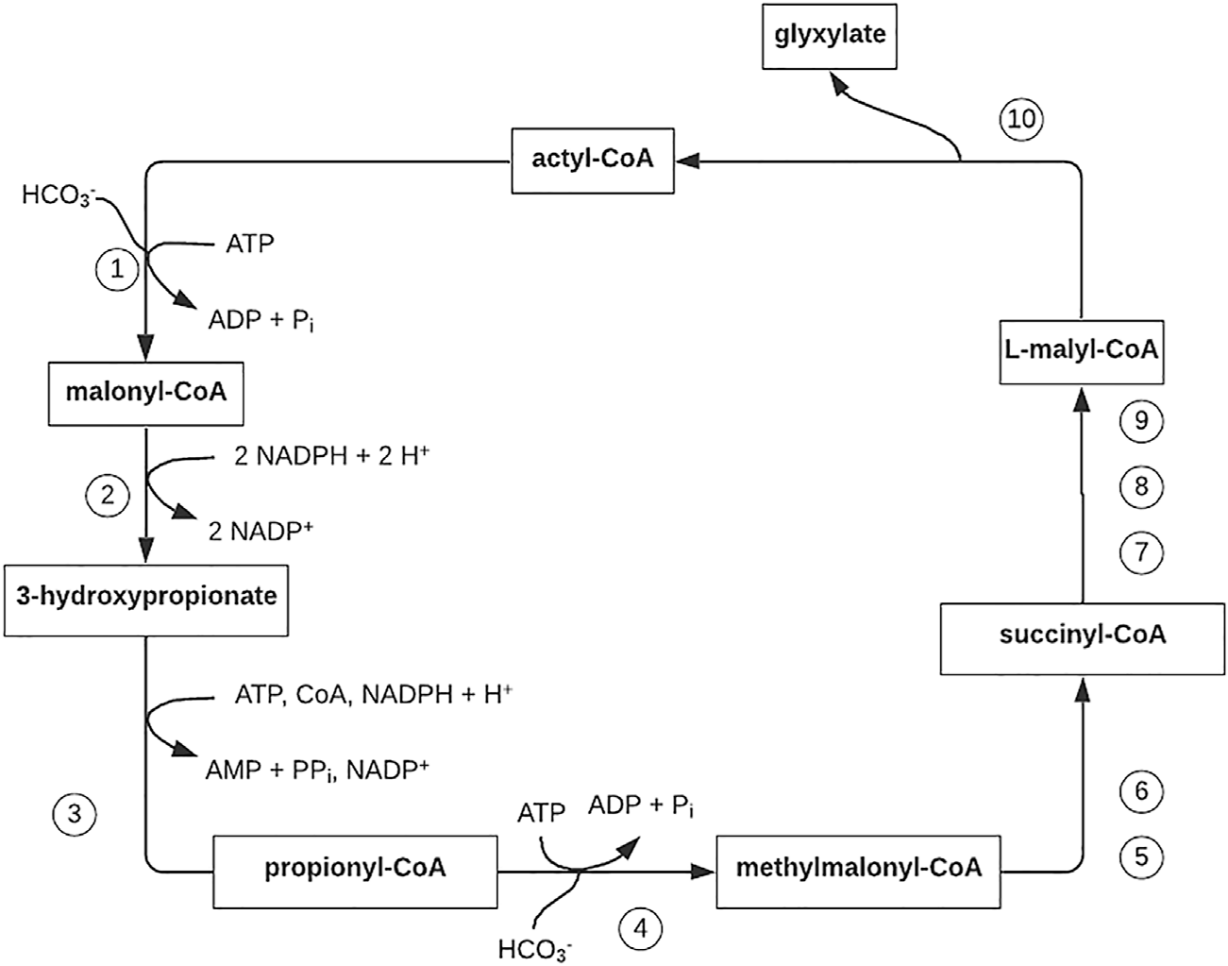
3-Hydroxypropionate cycle of autotrophic CO_2_ fixation in the phototrophic green non-sulfur eubacterium *Chloroflexus aurantiacus.* Figure redrawn after reference (Alber et al., 2008). Step 1, acetyl-CoA carboxylase; step 2, malonyl-CoA reductase (bifunctional; step 3, propionyl-CoA synthase (trifunctional); step 4, propionyl-CoA carboxylase; step 5,methylmalonyl-CoA epimerase; step 6, methylmalonyl-CoA mutase; step 7, succinyl-CoA–L-malate CoA transferase; step 8, succinate dehydrogenase (electron acceptor unknown); step 9, fumarase; step 10, L-malyl-CoA/β-methylmalyl-CoA lyase (bifunctional).

CO metabolism is linked to the global carbon cycle, which involves the oxidation of organic carbon to CO_2_ by heterotrophic organisms as an energy source and the replenishment of fixed organic carbon by autotrophic organisms in a reductive process called CO_2_ fixation. CO_2_ is returned to the carbon cycle by one of the following pathways (Ragsdale, 2004):

(i) The Calvin-Benson-Basham cycle,
(ii) The reductive tricarboxylic acid (TCA) cycle (Figure 4),
(iii) The 3-hydroxypropionate cycle (Figure 5),
(iv) The autotrophic CO_2_ fixation in reduction acetyl-CoA pathway or
(v) The Wood-Ljungdahl (acetyl-CoA) pathway.

Three types of CO metabolism are recognized: aerobic, anoxic, and anaerobic (Table 1). The aerobic matabolism involves exogenous electrons, while the anoxic uses the internal generation of intermediates as an electron acceptor (Diender et al., 2015). A relatively well-studied example of respiratory CO metabolism is CO oxidation coupled with oxygen reduction (Meyer and Schlegel, 1983). Oxygen microbes of the genus *Carboxydotrophic* use CO as a source of carbon and energy. They transfer electrons from CODH by catalyzing the oxidation of CO through the respiratory chain, which eventually reduces O_2_ according to the equation shown in Table 1. CO_2_ is assimilated as a source of cellular carbon *via* the Calvin-Benson Bassham pathway. These bacteria are well adapted to the role of CO detoxification in the environment as they are highly prone to CO uptake (Ragsdale, 2004).

**TABLE 1:** Carbon monoxide metabolism reactions (Diender et al., 2015).

Conditions	Metabolism	Reaction equation
Anaerobic	Hydrogenogenic	$CO+H_{2}O\to {CO}_{2}+H_{2}$
	Methanogenic	$4CO+2H_{2}O\to {CH}_{4}+3{CO}_{2}$
	Acetogenic	$4CO+2H_{2}O\to CH3{COO}^{-}+H^{+}+2{CO}_{2}$
	Solventogenic (ethanol)	$6CO+3H_{2}O\to C_{2}H_{5}OH+4{CO}_{2}$
Anoxic	Sulfate	$4CO+{SO}_{4}^{2-}+H^{+}\to 4{CO}_{2}+{HS}^{-}$
Aerobic	Oxygen	$2CO+O_{2}\to 2{CO}_{2}$

CO can also be converted to CH_4_ under anaerobic conditions by a range of microorganisms, including methanogenic archaea, as described in Section 4.1.2. These microorganisms use CODH, an enzyme that allows CO as a carbon source and its oxidation (Navarro et al., 2014). However, the efficiency of methanogenesis with CO as a substrate is not very high, and only three microorganisms have been marked as capable of producing CH_4_ from CO: *M. thermoautotrophicus, Methanosarcina acetivorans*, and *Methanosarcinabarkeri*. Most of these organisms use CO for growth and metabolizing in the Wood-Ljungdahl pathway (Ragsdale and Pierce, 2008).

**FIGURE 6:**
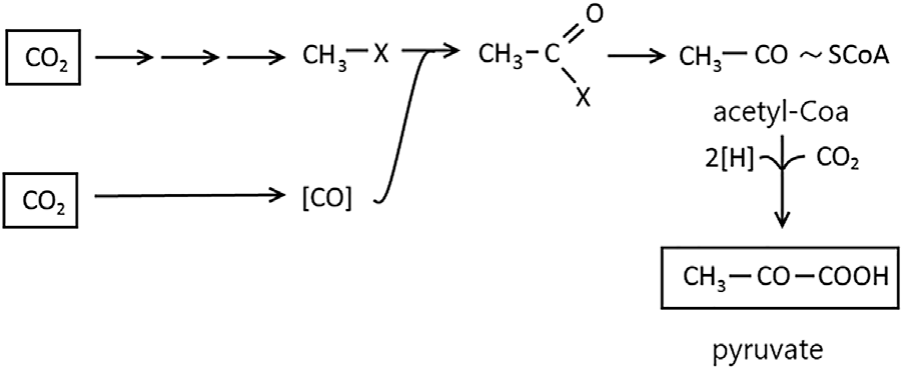
Autotrophic CO_2_ fixation in reduction acetyl-CoA pathway. Figure redrawn after reference (Schlegel, 2004).

Autotrophic CO_2_ fixation by methanogenic microorganisms, sulfate-reducing and acetogenic bacteria occurs without carboxylation phase reaction. The synthesis of cellular material from CO_2_ proceeds with the reductive acetyl-CoA pathway involving pyruvate. The reactions responsible for this process were detected using radioactive compounds and enzymatic studies with *M. thermoacetotrophium*. The mechanism is the reduction of CO_2_ to methanol in a bound form (Figure 6). The second CO_2_ molecule is reduced to CO by CODH. The reducing force is provided by the H_2_ activation by hydrogenases and transmitted by enzymes reacting with F420 (8-hydroxy-5-deazaflavin) or NADP (Nicotinamide adenine dinucleotide phosphate). As a result of the methyl-1x carbonylation, acetyl-X is formed, and the reductive carboxylation of acetyl-CoA by pyruvate synthase leads to pyruvate from which cell materials are formed *via* well-known pathways (Figure 6) (Schlegel, 2004).

### 4.3 CO microbiological production—Pathways and enzymes

The process of CO metabolism is much better described in the literature than the production, which is connected with researchers focus on CO utilization during fermentation process and bioethanol production (Abubackar et al., 2011). CO is biologically generated during the following pathways:

(i) The Wood-Ljungdahl (acetyl-CoA) pathway,
(ii) Conversion of S-methylthioadenosine to methionine (Dai et al., 1999),
(iii) Aromatic amino acid metabolism by bacteria (Hino and Tauchi, 1987),
(iv) Aldehyde decarbonylation by plants (Cheesbrough and Kolattukudy, 1984),
(v) Heme degradation by heme oxygenase (Tenhunen et al., 1969),
(vi) Homoacetate and acetate fermentation (Schlegel, 2004).

#### 4.3.1 Wood-Ljungdahl pathway

**FIGURE 7:**
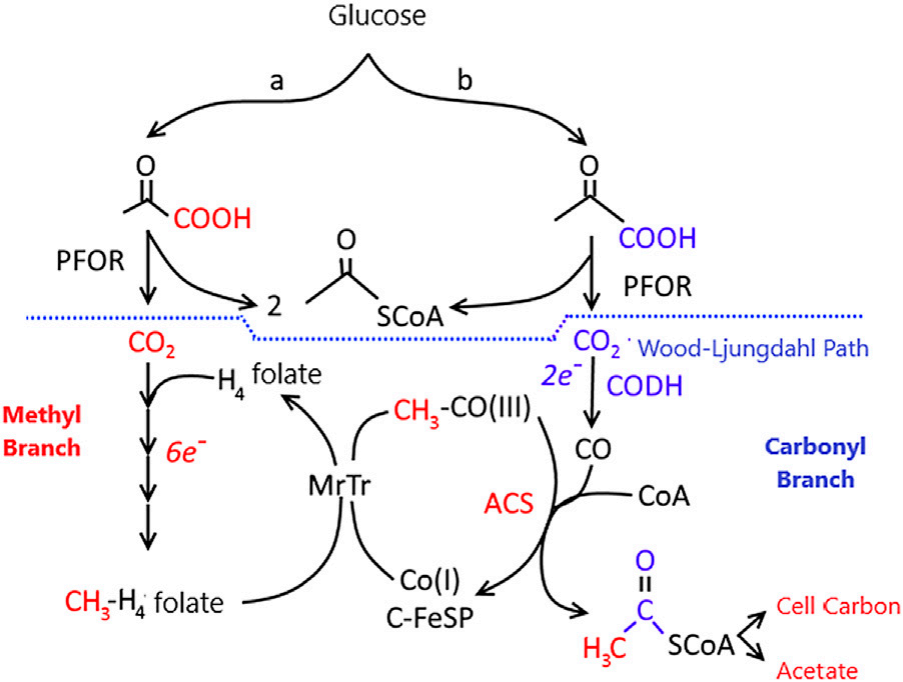
The Wood-Ljungdahl pathway of autotrophic CO and CO_2_ fixation. CODH, CO dehydrogenase; ACS, acetyl-CoA synthase; MeTr, methyltransferase; CFeSP, Corrinoid iron-sulfur protein; PFOR, pyruvate ferredoxin oxidoreductase. Letters **(A,B)** indicate two branches of the Wood-Ljungdahl pathway. Reactions leading to the formation of the methyl group of acetyl-CoA are colored red, while those leading to the carbonyl group are colored blue. Figure redrawn after reference (Ragsdale, 2004).

The Wood–Ljungdahl pathway (Figure 7) is found in a broad range of phylogenetic classes and is used in both the oxidative and reductive processes. The pathway is used in the reductive direction for energy conservation and autotrophic carbon assimilation in acetogens. When methanogens grow on (H_2_ + CO_2_), they use the Wood–Ljungdahl pathway in the reductive direction (like acetogens) for CO_2_ fixation (Ljungdahl, 1994). However, they conserve energy by the conversion of (H_2_ + CO_2_) to CH_4_ (Stupperich et al., 1983).

Organisms using the reduction pathway of the acetyl-CoA cycle, referred to as the Wood-Ljungdahl pathway, reduce atmospheric CO_2_ to CO through dehydrogenase (CODH), with Ni, Zn, Fe cofactors (Menon and Ragsdale, 1999) (Figure 7). The electron donor for this reaction is hydrogen. The CO combined with the dehydrogenase is linked to a methyl group carried by a corrinoid protein with a structure similar to vitamin B12. This protein takes methyl groups from tetrahydro-methanopterin and attaches to enzyme-bound CO. The acetyl group formed in the reaction is transferred to coenzyme A, which leads to the formation of acetyl coenzyme A (Ragsdale, 2008).

Unlike the Calvin cycle reducing the TCA pathway or the 3-hydroxypropionate cycle, the Wood-Ljungdahl pathway consists of two branches that require eight reducing equivalents and one ATP (adenosine triphosphate) to form acetyl-CoA from the two CO_2_. ATP energy is recovered by phosphorylation at the substrate level during acetate formation, but net ATP is not obtained, requiring an anion driving force for net energy conservation (Diender et al., 2015).

#### 4.3.2 Conversion of S-methylthioadenosine to methionine

**FIGURE 8:**
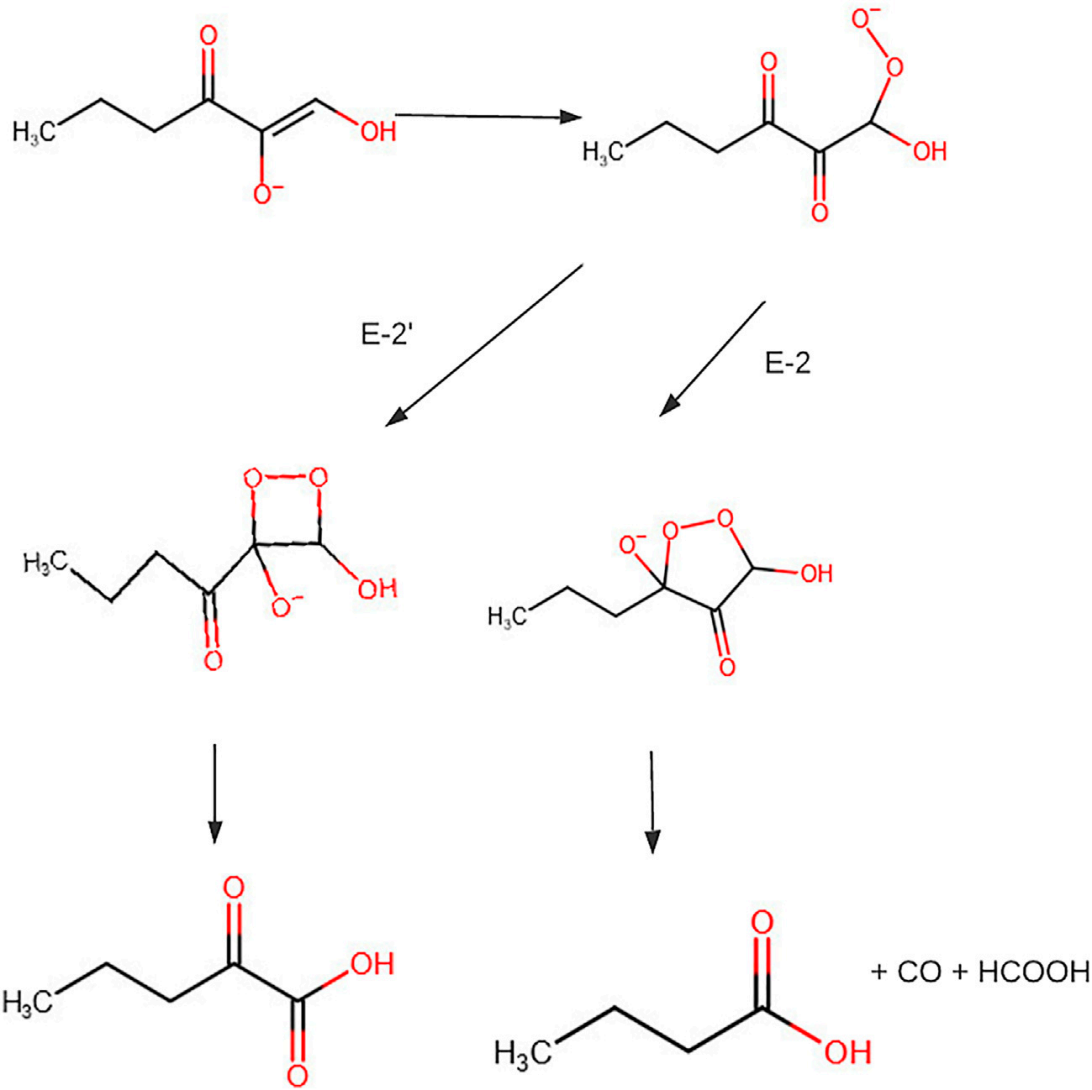
Conversion of S-methylthioadenosine to methionine. Figure redrawn after reference (Dai et al., 1999).

Previous isotope tracer studies of purified E-2 and E-29 activity in extracts and experiments for CO production led to proposing a mechanism that could head to the formation of two different sets of products (Figure 8). The hydroperoxide radical or anion adds to C-2 or C-3. The addition to C-3 produces formate, CO, and butyrate. The addition to C-2 produces formate and 2-oxopentanoic acid. The metal ion affects the active site structure and thereby determines the point of addition of the hydroperoxide radical or anion and, consequently, the nature of the products (Dai et al., 1999).

#### 4.3.3 Homoacetate fermentation

Some *Clostridium* bacteria transfer the hydrogen equivalents released in the early stages of substrate oxidation, converts CO_2_ to acetate with the following formula:

$$8\left[H\right]+{CO}_{2}\to {CH}_{3}-COOH+2H_{2}O$$ (1)

**FIGURE 9:**
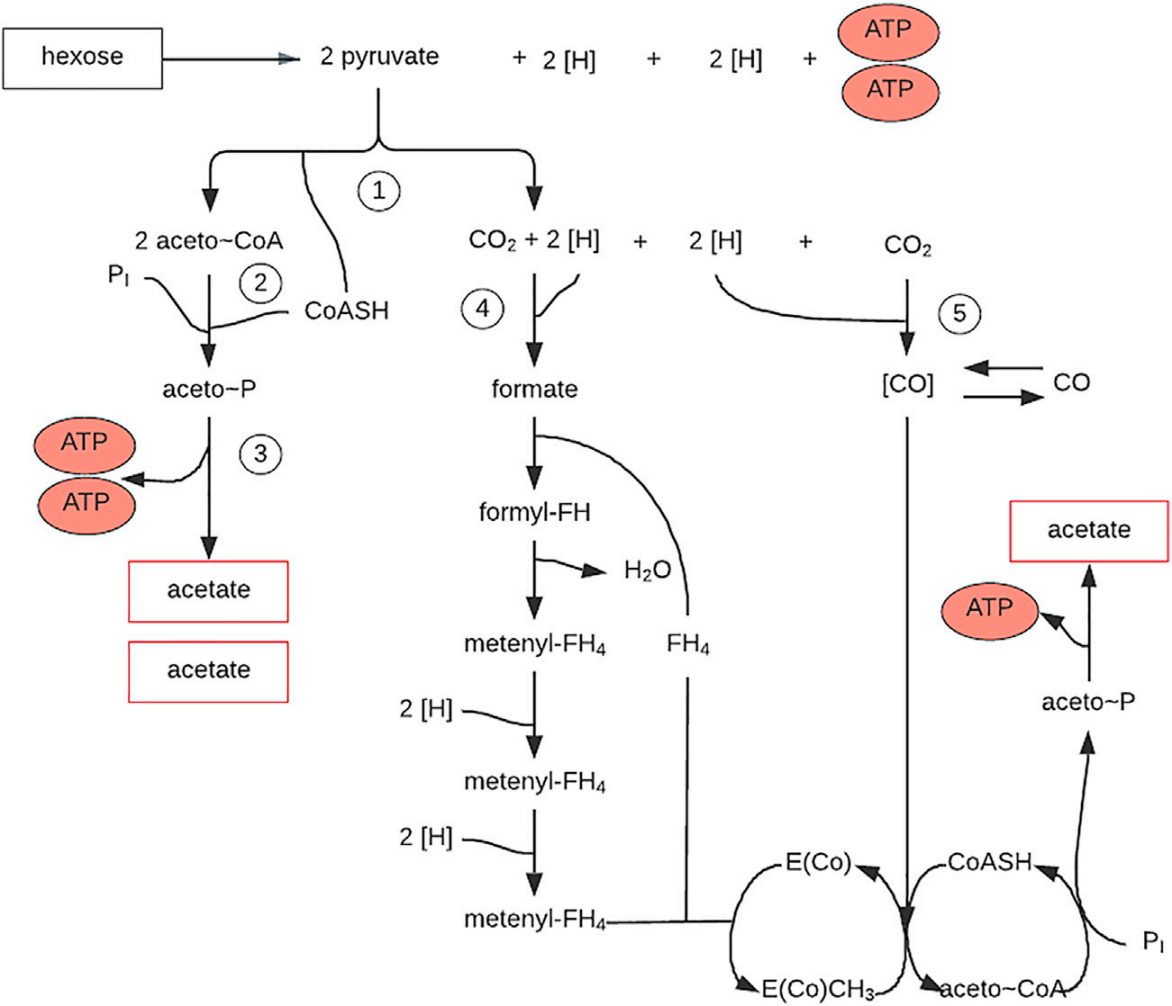
Acetate biosynthesis pathway from hexose (*via* the Acetyl-CoA pathway) in *Clostriudium thermoaceticum*. E (Co), corrinoid protein; FH4, tetrahydrofolic acid; (H), hydrogen equivalents in the form of NADH_2_ or FdH_2_; CO, exogenous carbon monoxide; (CO), bound carbon monoxide. Figure redrawn after reference (Schlegel, 2004).

The thermophilic bacteria *C. thermoaceticium* and the mesophilic *C. formicoaceticum* ferment glucose primarily into acetate. They metabolize hexose in the fructose bisphosphate pathway, producing nearly 3 moles of acetate for every mole of glucose used. A large proportion of the CO_2_ generated during pyruvate decarboxylation must be rebound to hydrogen acceptor to achieve this. The formation of acetate from CO_2_ and reducing equivalents (electrons) obtained in the initial oxidation reactions proceeds according to the diagram (Figure 9). Hexose is converted to pyruvate in the fructose bisphosphate pathway. Two enzymes—pyruvate oxidoreductase and ferrodoxin—are involved in forming acetate, CO_2_, FdH_2_ (α_2_β_2_ enzyme), and ATP from pyruvate. CO_2_ serves as a hydrogen acceptor. Partly it is reduced by formate dehydrogenase to formate, then to the methyl group of the third acetate molecule, and partly by CODH to CO, which is the acetate carboxyl group (Schlegel, 2004).

#### 4.3.4 Acetate fermentation

CO_2_ is reduced to CO (with CODH enzyme) in the acetate fermentation pathway, which finally gives the carboxyl group of acetate; methyltetrahydrofolate is carbonylated and acetyl-CoA and finally acetate is formed (Gottschalk, 1986). That reaction is, therefore, reversible under physiological conditions:

$${CO}_{2}+X-H_{2}↔CO+X+H_{2}O$$ (2)

**FIGURE 10:**
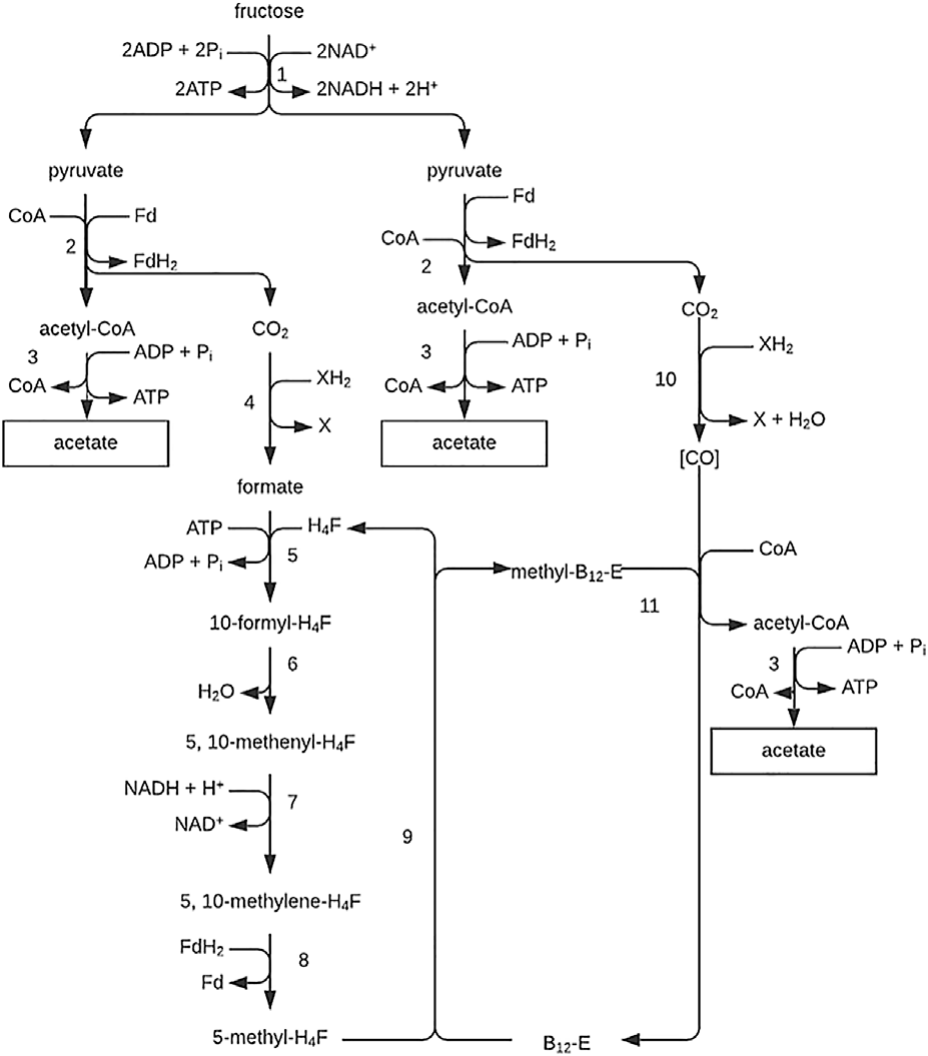
Pathway of the acetate fermentation. Figure redrawn after reference (Gottschalk, 1986). 1, Degradation of fructose *via* the Embden-Meyerhof-Parnas pathway; 2, pyruvate-ferredoxin oxidoreductase; 3, phosphotransacetylase plus acetate kinase; 4, formate dehydrogenase; 5, formyl-tetrahydrofolate synthetase; 6, methenyl-tetrahydrofolate cyclohydrolase; 7, methylene-tetrahydrofolate dehydrogenase; 8, methylene-tetrahydrofolate reductase; 9, tetrahydrofolate: B 12 methyltransferase; 10, CO dehydrogenase; 11, acetyl-CoA-synthesizing enzyme (probably ATP-requiring); (CO), enzyme-bound.

As depicted in Figure 10, the pathway also allows outlining the routes used for acetate formation from CH_3_OH + CO_2_ and H_2_ + CO_2_. The strategy is to make CO from CO_2_ and methyltetrahydrofolate from H_2_ + CO_2_ or methanol. Thus, part of the methanol has to be oxidized to reduce CO_2_ to CO; methyltetrahydrofolate and CO finally yield acetate.

### 4.4 CO production during biological waste treatment processes

The growth in the human population combined with industrialization, urbanization, and improving living standards increases the amount of generated waste (Singh et al., 2014). It is estimated that global waste mass will increase to 3.4 billion tons in 2050 (Karim et al., 2019). Organic waste, such as kitchen and food waste, garden waste, agricultural and animal wastes, and sewage sludge, deserves special attention due to their link to greenhouse gas (GHG) emissions, odor, and sanitary and human health concerns. Grass, leaves, branches, and household food waste, collectively referred to as “bio-waste” (or “biowaste”), make up the largest share of municipal solid waste in low- and middle-income countries. Moreover, researchers and community organizers note that about one-third of the organic waste produced globally is food waste (Bellemare et al., 2017), and the amount is still increasing. Therefore, both the production and management of organic waste are perceived as an environmental problem globally.

An inherent element of organic waste management is the emission of air pollutants, which include GHG (the most important of which are CH_4_, CO_2,_ and N_2_O), ammonia, volatile organic compounds (VOCs), and hydrogen sulfide (Rincón et al., 2019). These gaseous emissions have been widely noted in the literature (Cao et al., 2019). On the other hand, production of CO during biological waste treatment processes—according to the bibliometric analysis—seems to be mostly ignored. We have been proposing that more attention should be paid to CO generation, fate, emissions, and its potential synergistic opportunities for a more sustainable development, which in case of CO—can change the term “pollutant.”

#### 4.4.1 Composting process

In Europe, composting is one of the dominant bio-waste treatment options. Of the total of 48 million tonnes processed at ∼4,250 plants, more than 30.5 million tonnes (>60%) were processed in 3,400 composting plants in 2019. Additionally, 4.4 million tonnes of bio-waste is integrated into composting and anaerobic digestion plants (European Compost Network e.V., 2019). Directing bio-waste to composting is the leading practice in almost all European countries (except Sweden and Denmark).

Data on the production (generation) of CO during the waste composting are minimal. There are few reports in the literature about CO sources, CO formation mechanisms, and optimal CO emissions conditions (Hellebrand, 1998; Hellebrand and Kalk, 2001; Haarstad et al., 2006; Hellebrand and Schade, 2008). The discovery of high CO concentrations (∼100 ppm) during the biological decomposition of OM was surprising at the time. Back then, CO was known as an incomplete combustion product but not composting (Hellebrand, 1998). Nevertheless, follow-up studies have shown that biomass’s gradual decomposition leads to O_2_ depletion and CO release (Arshadi and Gref, 2005). CO can reach significant levels when composting waste exceeding 1,000 ppm (Haarstad et al., 2006; Stegenta et al., 2018; 2019b). CO emissions are also a secondary source of GHG emissions from the composting process, especially related to such substrates as green waste, animal and municipal waste (Andersen et al., 2010a; Sánchez et al., 2015). Due to the health effects of CO on humans and the legal requirements for the hermetization of composting facilities, the process and the associated CO production may also pose a risk to composting plant workers directly involved in handling the process (Sobieraj et al., 2021).

CO generation has been observed during composting of green waste (Stegenta et al., 2019b), a mixture of green waste with dairy manure (Hellebrand and Kalk, 2001), organic waste (Haarstad et al., 2006), and during aerobic biostabilization of the municipal waste (Stegenta et al., 2018). The knowledge obtained so far has allowed for the formulation of two hypotheses on the mode of CO production. CO formation has both abiotic and biotic nature, which was determined not only based on observations of CO formation during the process but also during the analysis of samples of sterilized and non-sterilized material subjected to composting (Stegenta-Dąbrowska et al., 2019). Thus, CO production from waste composting is seen as a combination of physical processes dependent on temperature and O_2_ concentration and related to microorganisms’ biological activity (Sánchez et al., 2015).

The most common observation in research on gas emissions from composting is that CO production increases immediately after the start of the process, both on a laboratory, pilot, and industrial-scales (Stegenta et al., 2018; Stegenta et al., 2019a), and it subsequently declines, often quite sharply (Stegenta et al., 2018). Modeling of CO production during the typical 14-day composting process showed, that its concentration can reach 3.2% (31,600 ppm) and 36.1% (360,000 ppm) for reactors with the daily release of accumulated gas and without ventilation, respectively (Sobieraj et al., 2021). High CO production in the initial stage of the process correlates with the temperature increase in the compost piles and shows the most significant increase in thermophilic conditions (Stegenta et al., 2019a). Interestingly, the temperature appears to be the driver of CO as well. For example, the second CO peak production was observed after as many as 100 process days was caused by the 80°C spikes (Andersen et al., 2010a). Higher CO concentrations were recorded mainly for sterile material compared to non-sterile samples (Stegenta-Dąbrowska et al., 2019). Due to the dependence of CO emissions on temperature, low production rates of CO are also observed in winter piles when the ambient temperature is low (<0°C, December—March in Europe) (Stegenta et al., 2019a).

The increased O_2_ availability, together with the temperature increase, stimulates CO production (Phillip et al., 2011). This was also confirmed experimentally on a technical scale (Stegenta et al., 2018). During the aerobic biostabilization of municipal waste, higher levels of CO were recorded in perforated reactors, in which the oxidation of the waste was higher compared to the tightly sealed material. Based on the reports on the stimulating effect of temperature and O_2_ on CO production, it was determined that the CO source’s thermochemical processes have a dominant influence, and O_2_ is playing a slightly lesser role (Stegenta-Dąbrowska et al., 2019). It also proves that the thermal degradation of OM in waste, resulting in CO production, may occur at a relatively low temperature, not exceeding 100°C (Stegenta-Dąbrowska et al., 2019).

Microorganisms are also assumed to be responsible for CO production, especially in aerobic conditions. Their influence as suppliers of substrates and accelerating CO production under aerobic conditions was proposed (Rich and King, 1999), specifically, the oxidation of fatty acids and free radicals’ breakdown leading to humic substances. On the other hand, the mesophilic conditions (∼40°C) may favor biogenic CO production (Stegenta-Dąbrowska et al., 2019). There are also reports of anaerobic CO production in unsterilized samples. Transient peaks of increased CO concentration were observed, resulting from a temporary O_2_ depletion (Haarstad et al., 2006). The activity of methanogenic bacteria explained the production of CO under such conditions. However, the mechanisms of aerobic and anaerobic CO production are still subject to speculation (Stegenta-Dąbrowska et al., 2019).

The decrease in CO production observed in the later stages of the composting process is explained by achieving the maximum growth of microorganisms that consume the O_2_ necessary for the thermochemical oxidation of CO (Stegenta-Dąbrowska et al., 2019). Faster reduction of O_2_ was observed at higher temperatures (50°C–60°C), which proves the occurrence of optimal thermal conditions for microorganisms active in the composting process (Stegenta-Dąbrowska et al., 2019). These observations are additionally confirmed by the observed increase in CO_2_ concentration, especially in non-sterilized material subjected to aerobic processes (Phillip et al., 2011).

The inverse correlation between CO and CO_2_ concentration highlights the likely oxidation of CO by bacteria (Phillip et al., 2011). The highest concentrations of CO_2_ occurred in a wide temperature range; during the experiment on non-sterile material (Stegenta-Dąbrowska et al., 2019), its maximum production was recorded at 40°C, consistent with the results of (Lee et al., 2012). On the other hand, in studies conducted by (Eklind et al., 2007), the highest emission occurred at higher temperatures, overlapping the previously mentioned minimum O_2_ concentration (50°C–55°C). At 65°C, CO_2_ was further produced, consistent with the range of activity of CO metabolizing bacteria (55°C–82°C) (Stegenta-Dąbrowska et al., 2019).

This sometimes conflicting evidence indicates the composting process’s complexity and its dependence on various (mainly) biodegradable substances contained in the material. Moreover, during the composting process, CO becomes an energy source for anaerobic carboxydotrophic bacteria, which contributes to reducing CO concentration (Pomaranski and Tiquia-Arashiro, 2016). As proved by (Hellebrand and Kalk, 2001), the overlapping of both types of processes, CO oxidation and its consumption by microorganisms, causes variability in CO emissions and may lead to several CO release spikes during waste composting. The presence of biological determinants of CO formation was also confirmed in the case of non-sterilized material, specifically the importance of process time on net gas production (Stegenta-Dąbrowska et al., 2019). This factor may indirectly affect the growth kinetics of microorganisms and thus the formation or metabolism of CO.

The net CO emission rate depends on competitive processes of production and microbial oxidation, with each of these processes being mainly influenced by the process temperature and O_2_ concentration. Due to the dual—biotic and abiotic—nature of CO production, the factors affecting its formation are also, among others, moisture content of composted material or the presence of other gases (Stegenta-Dąbrowska et al., 2019). Moreover, the CO production is influenced by the substrate’s composition, including OM content (Phillip et al., 2011). However, the number of sources mentioning these variables is limited in the literature; there are also no experiments analyzing these factors.

The moisture content of the material was taken into account by (Hellebrand and Schade, 2008), according to which the CO production is dependent on the mutual interaction of O_2_ and water content, and the decrease in CO production is probably the result of drying out of the decomposed material. This is in line with reports by (Schade, 1997), showing that high CO production in the early stages of the process is because, initially, the samples are wet, and the O_2_ has not yet been consumed. The laboratory analyzes carried out by (Haarstad et al., 2006) showed that the addition of lime to aerobic processes causes a significant increase in the CO concentration of CO (average value of 101 and 486 ppm without and with the addition of lime, respectively). This is explained by the supply of a high load of OM and thus faster O_2_ depletion, which is also confirmed by the overlap of the CO production peak with a strong O_2_ decrease and an extended presence of CH_4_. Moreover, in the same experiment, a strong correlation of CO with H_2_S was noted during anaerobic degradation. This experiment thus confirms the above-mentioned hypothesis of CO production by methanogens (Rich and King, 1999).

#### 4.4.2 Spatial distribution in composting waste pile

The subject of the spatial distribution of CO in composted waste is rarely undertaken by researchers. Its distribution in the material shows high variability, both in the cross-sections and longitudinal sections of the piles, and may depend on the scale of the process and its management method, the type of substrates, and environmental factors (Stegenta et al., 2019a; Stegenta et al., 2019b). Nevertheless, the increased CO concentration was observed in the entire cross-section of the material shortly after the pile was formed, and the maximum concentrations occurred earlier than for other gases (Stegenta et al., 2019b).

An O_2_ gradient influences the spatial distribution of CO concentration in the compost pile—an increased CO concentration occurs in areas with high oxidation. This is due to the highest CO concentrations near the top of the stack, while the lowest CO content is characteristic of its lower part, in line with the passively aerated waste stacks/piles (Andersen et al., 2010b). Similarly, a higher CO concentration was recorded at the beginning and end of the pile prism, which may be associated with a larger contact surface of the material with ambient air (Stegenta et al., 2019b). Additionally, due to the material’s anaerobic zones, an increase in the CO content was observed after the material was turned over (Hellebrand and Schade, 2008), i.e., a typical practice in industrial scale composting.

However, the reason for the apparent compost pile sections with high CO and O_2_ concentrations is not clear. The high CO levels were present in the pile’s surface layer, but the O_2_ concentrations were low (Stegenta et al., 2019a; Stegenta et al., 2019b). Additionally, CO was not detectable as the oxidation increased. The O_2_ reduction with the pile depth led to the formation of anaerobic conditions in its core (<2% O_2_) during the composting of green waste and manure (López et al., 2016). This, in turn, favored the CO presence. CO concentration increased with the depth of sampling, reaching a maximum close to 800 ppmv at 80 cm depth. CO was also detected in the center of leaf and grass clippings piles (Hellebrand and Schade, 2008). This inverse CO dependence on O_2_ also manifested itself indirectly in CO concentration changes depending on the wind direction (Andersen et al., 2010b). Higher CO levels were recorded on the west side for the east-to-west wind and not on the east side, where higher O_2_ concentrations were observed.

The decomposition of CO in the composted mass of waste was also dependent on the temperature, and its increase caused an increase in the CO release rate (Phillip et al., 2011). Therefore, the optimal conditions for CO production are shaped by the thermal “chimney effect” in compost (Andersen et al., 2010b). In this way, areas with increased CO concentration overlap with thermophilic zones in the composted mass (Stegenta et al., 2019b). There was also an inverse correlation between the CO and CO_2_ concentrations in a pile. The minimum CO concentration occurred as soon as CO_2_ reached its maximum, and when CO is present at high concentrations, the level of the CO_2_ decreases (Stegenta et al., 2019a). This is likely related to the CO consumption by microorganisms, which results in CO_2_ production (Hellebrand, 1998).

#### 4.4.3 Biomass and bio-waste storage and transportation

Dangerous CO levels and reduced O_2_ concentrations were also identified during biomass and waste storage, e.g., wood pellets, forest residues, liquid pig manure, and dry grain (Whittle et al., 1994; Svedberg et al., 2004; He et al., 2012; Matulaitis et al., 2015). Laboratory analyses of gases emitted from the storage of various biomass types have shown that the CO concentration in reactors’ headspace increases with time (Kuang et al., 2008). A faster accumulation rate was recorded at the beginning of trials, and after a few days, the CO emissions stabilized, following the first-order reaction kinetics.

Occupational accidents related to the maritime transport of wood pellets were researched (Svedberg et al., 2008). CO concentration in the sealed containers and shipping vessels can reach lethal levels ranging as high as 1,460–14,650 ppm and diffuse into adjacent spaces within the first week of wood pellets storage. The high CO production at the beginning of organic materials’ storage is consistent with (Kuang et al., 2009) observations.

The ratio of headspace/reactor volume (H/R) is a significant determinant of the emission rate and CO concentration. At high H/R ratios, high peak emissions and reaction rates were reported. The net CO production depended on the O_2_ concentrations, i.e., greater O_2_ availability for pellets’ oxidation results in the CO release. However, the higher H/R makes slows down the decomposition of biomass as excess air/O_2_ utilization takes time. Other factors on CO emissions from waste and biomass storage, such as temperature and material moisture content, were the subject of additional research (He et al., 2012). The increase in temperature caused an increase in CO concentration up to 1,600 ppm. At lower moisture, the CO decreased at the expense of CO_2_ production. For this reason, CO production is likely the result of a combination of chemical and biological processes.

#### 4.4.4 Anaerobic processes

The CO emergence during the anaerobic digestion process is related to the activity of the CODH. It has been discovered in most of the methanogenic and acetogenic bacteria and used in catabolic and anabolic oxidoreductase reactions (Zeikus et al., 1985). High CODH levels have been observed in *Methanothrix soehngenii*, one of the major species responsible for acetate catabolizing in fermentation systems (Kohler and Zehnder, 1984) and in sulfate-reducing bacteria (Schauder et al., 1986). CO was produced in the presence of 80% H_2_ and 20% CO_2_ gas mixture by *M. thermoautotrophicum* (Conrad and Thauer, 1983) and *M. barkeri* by culturing both pure and enriched acetate cultures (Hickey, 1987). In the case of the second of these strains, CODH constituted approx. 50% of the soluble bacterial protein (Krzycki and Zeikus, 1984). CO is also an important component of the acetate-to-CH_4_ conversion performed by *Methanosarcina* strain TM-1 and *M. acetivorans* (Nelson and Ferry, 1984). However, analyses carried out on methanogens lacking this enzyme indicated that they did not produce CO in the batch culture at a detectable level (Bott et al., 1985).

During the process of methanogenesis, the decomposition of acetate into bound carbonyl and methyl intermediates leads to the subsequent oxidation of the former with CODH to CO_2_, with the simultaneous production of reducing equivalents used for the reduction of methyl coenzyme M (methyl-CoM) to CH_4_ (Kohler and Zehnder, 1984). These processes excluded free CO as used carbonyl intermediates (Eikmanns and Thauer, 1984). CO equilibrates with a component of the carbonyl pathway (Nelson and Ferry, 1984), and the concentration of this component (carbonyl or bound CO, likely to metal) is directly related to the acetate concentration during its methanogenesis (Hickey et al., 1987). This conclusion was reached while observing a CO increase after adding acetate as a substrate during system equilibrium-focused work (Hickey et al., 1987). A similar trend was noted in another experiment when the acetate accumulation was associated with a proportional increase in CO gas (Hickey and Switzenbaum, 1991). In turn, approx. 54% of the energy available for the CH_4_-to-acetate conversion was used to oxidize CO to CO_2_ in fermentation chambers operating under mesophilic and thermophilic conditions (Hickey and Switzenbaum, 1990). To the contrary, the relationship between CO and acetate has not been noted by (Bae and McCarty, 1993). Together with (Hickey and Switzenbaum, 1990) they explain this by the different conditions of the anaerobic fermentation process and the CO production and consumption by various bacterial strains, which may result in the observed differences and the lack of apparent trends. The microbial flora is a complex system, and their mode of operation change over time, which may also translate into other pathways of compound degradation and the potential for CO production.

The increase in the CO concentration with the increase of H_2_ concentrations in addition to the dependence of CO on acetate was observed in anaerobic fermentation (Bae and McCarty, 1993). The authors explain this possibility of CO production by methanogens using H_2_ or acetate as an intermediate product of the metabolic pathway or in the form of an electron sink product. Additionally, the literature reported increasing the organic load in the process on CO and CH_4_ concentrations. The CO concentration increased, resulting in a decrease in CH_4_ (Hickey and Switzenbaum, 1990). A relationship between CO concentration and the accumulation of volatile fatty acids (VFAs) in the liquid-phase of fermentation has been reported by (Molina et al., 2009). An increased CO and H_2_ levels in the gas-phase is a typical sign of organic overload (Huang et al., 2000). The fact of the increased CO and H_2_ levels and the negligible presence of CH_4_ is related to the imbalance between acidogenic and methanogenic processes (Ahring et al., 1995).

The importance of CODH and practical implications increased due to the latest findings on its biological mediation of water-gas shift bioreaction (BWGS) (Alfano and Cavazza, 2018). This process involves converting CO to H_2_ according to:

$$CO+H_{2}O\to H_{2}+{CO}_{2}$$ (3)

Apart from using the critical enzyme CODH, reaction (3) is based on the activity of the dihydrogen-producing enzyme [NiFe]-hydrogenase (Kung and Drennan, 2011). H_2_ production during the reaction (3) is assisted thermodynamically. WGS requires appropriate conditions such as low temperature and pressure and darkness (Bičáková and Straka, 2012). The use of CODH-producing microorganisms and the ability to convert CO to H_2_ at room temperature and pressure make them a promising alternative to inorganic industrial processes (Alfano and Cavazza, 2018). The biological reaction of WGS may become a favored technology for biohydrogen production not only from an ecological but also an economic point of view (Rittmann et al., 2015). Due to the processes taking place at an ambient temperature and atmospheric pressure, they can be carried out locally, using available bio-waste materials. The production of H_2_ from readily available biomass and bio-waste will reduce the costs of substrate transport and energy (Bičáková and Straka, 2012). Therefore, the controlling of CO production during biological OM decomposition is crucial.

## 5 Future directions and limitations

With reference to the above information about BWGS, it is necessary to analyze the possible future directions of the development of CO extraction from biological waste treatment processes and the factors that may limit them. The coupling of composting technology and CO production at industrial scale may face many obstacles for which no solutions are currently being developed.

First, there is a need for a thorough understanding of the mechanisms of CO production during aerobic organic waste treatment processes. The existing premises assume that it occurs in biotic way with the participation of microorganisms, but to date, no specific groups of bacteria that carry out this process have been indicated. This is important due to the fact that the researchers isolated aerobic strains capable of metabolizing CO and at the same time noticed the presence of anaerobic bacteria in compost piles (see Section 4.1). As this topic is currently not addressed by researchers, it is necessary to plan comprehensive basic research based on microbiology and molecular biology, taking into account not only isolation and identification of the bacterial species, potentially responsible for the CO production, but also the analysis of expression of CODH encoding gene at different composting conditions. Identification of specific groups of microorganisms that are able to produce CO in the composted mass of waste will allow to control and adjust the process to optimal conditions conducive to their development, while taking into account the quality of the final product. Additionally, due to the observations of high CO production at the beginning of composting and its subsequent decrease, analyzes of the variability of microorganisms during the process should also be carried out; it is possible that the production of CO takes place through the cooperation of various groups of bacteria, and the disappearance of some due to the occurrence of unfavourable conditions causes a decrease in the activity of the strains correlated with them. Indirectly, it can be also drawn on the knowledge acquired in the BMWGS processes; while analyzing this reaction, a problem related to gas-to-liquid mass transfer was discovered (Alfano and Cavazza, 2018). It has been proven that the activity potential of CO-metabolizing microorganisms in BMWGS reactors depends on the concentration of CO, and due to slow diffusion from the bulk gas into the pores of the catalyst, the reaction rate is significantly reduced, as the organisms have to wait for the next part of the substrate (Amos, 2004). It may also translate into the composting process, in which the delivery of the CO by specific strains for subsequent groups of microorganisms is too slow, which results in the gradual death of the latter.

What is more, learning about specific bacterial strains may also lead to the development of precise protocols and recommendations for conducting composting directed at CO production, regarding e.g., the use of a specific dose of microorganisms added to compost piles or bioreactors at the right phase in the processes of biological oxygen degradation of OM, as well as the method of their application. Such a solution can also lead to the development of another niche, dealing with the production and distribution of ready-made biopreparations dedicated to such composting processes, excluding groups that offset each other.

Taking into account the currently achieved CO concentrations from the composting process, close to 1,000 ppm on a technical scale, it is also necessary to intensify the production of this gas, so that its generation rate is valuable for further processing in order to obtain specific products on a semi-industrial or industrial scale. However, this intensification will be possible when all process conditions influencing the biological formation of CO are known; on their basis, it will be possible to develop a model that will take into account the most important variables of the process, including the effect of O_2_ concentration and temperature. This is particularly important in the context of reports on the more efficient metabolism of CO by bacteria during BMWGS conducted at high temperatures, which results from increased gas diffusion rates (Alfano and Cavazza, 2018). Conducting the process with specific parameters adjusted to this scenario will allow to maximize the yield of CO.

As mentioned earlier, composting is one of the dominant organic waste treatment methods used in Europe; the number of currently operating plants of this type provides extensive technical facilities that could serve the purposes of the future development of infrastructure focused on CO production. However, it is necessary to develop solutions enabling the collection, transmission and storage of CO from the composting process, as well as specially dedicated bioreactors, enabling an effective process of OM degradation. These efforts to develop technologies for larger-scale work, however, entail high investment costs, which in turn highlights the need to finance pilot operations. However, in order to produce a valuable product, not only the availability of technological lines, but also the quality of the substrates fed to the composting process play an important role. Organic waste, covering a wide group of fractions of various origins, is a material with high variability, including seasonal one, consisting, among others, in different diet habits throughout the year (availability of vegetables and fruit, ending up in food waste) or resulting from different weather conditions (composition green waste from parks or gardens contains more fractions of leaves or grasses depending on the care treatments carried out during the year). Stable CO production from the composting process therefore needs to take into account this variability in order to produce a high amount of homogeneous product. This is related to the aforementioned modelling of CO production during the composting process; in the model of its intensification, it is necessary to reduce this variability to the basic properties, i.e., to take into account the influence of material moisture or OM content. In addition, in order to develop the technology of coupled composting and CO production, it is necessary to develop and implement an efficient system for collecting, storing and transporting the often dispersed waste stream to processing sites. Only with appropriate logistics will it be possible to continuously produce CO, competing with industrial inorganic processes.

As discussed earlier, the production of CO from composting processes is now recognized as a combination of biotic and abiotic processes but with an unknown ratio of both. While abiotic processes can be triggered by manipulating process parameters, controlling the activity of microorganisms is more challenging. One of the most serious obstacles to biological CO production during waste composting may be the effect of the CO itself on the microorganisms present in the composted mass. Due to the CO toxicity, there is a concentration barrier in the liquid phase that limits the growth of bacteria. This is based on the high affinity of CO to metalloenzymes that can completely block the catabolic activity of microorganisms (Alfano and Cavazza, 2018). For this reason, it is necessary to collaborate with omics data specialists and bioinformatics. Multidisciplinary collaborations can facilitate developing predictive models taking into account the variables influencing the metabolic processes of microorganisms. This, in turn, will allow to control and optimize the outcomes. In addition, the engineering of bacterial strains isolated from composted waste, which will adapt them to work with a gaseous substrate in a challenging environment, is also gaining importance in this context.

The current requirements for composting plants included in the BAT (best available technologies) reference indicate that these plants must implement procedures minimizing the impact of the process on the environment, mainly in terms of pollutants emitted to the atmosphere (Pinasseau et al., 2018). For this reason, one of the requirements is hermetization of compost halls, using negative pressure, limiting the leakage of unwanted substances outside. Taking into account the previously mentioned information on excessive CO production in bioreactors (both without ventilation and those opened to release process air), the safety aspect of composting plant employees becomes important. Having direct contact with composted waste, they are exposed to CO, the concentration of which significantly exceeds the acceptable generally limit values indicated by the WHO (Sobieraj et al., 2021). Therefore, it is necessary to develop personal protective equipment for workers currently working with the aerobic processing of organic waste in composting plants; however, it is also important to consider this problem in future facilities that interconnect composting plants with lines for further processing of the obtained CO, especially due to the intensification of this gas formation during the waste composting stage.

The coupling of composting and CO production processes may also face legal problems. The lack of regulations in this area, regarding the definition of the final product and the process itself, in relation to the applicable regulations, may become a barrier preventing the use of the produced CO and its circulation on the market. For this reason, it is important to conduct research in this area at the same time and involve other stakeholder groups, including representatives of the legislation.

It is also worth emphasizing that the potential for CO production from bio-waste composting is not negated but rather ignored. This is due to greater technology readiness of existing, competing methods such as syngas fermentation or BMWGS, which are still under development. It should be emphasized, however, that competing methods do exist, but in this aspect it is not only about economic efficiency, but about searching for new ways of circularity. Additionally, as with other technologies, biological processes based on microorganisms activity during composting occur at ambient temperature and pressure, lowering energy requirements and costs of CO production.

## 6 Conclusion

This review analyzed the literature on the subject of CO production during the processes of aerobic and anaerobic biological waste treatment, showing that the current state-of-the-art lacks comprehensive studies of the conditions under which CO is formed during composting. The mechanism of CO generation from this process is also unexplained. The impact of the type of substrate on the amount of CO emissions has not been investigated here; factors influencing CO formation and process parameters such as waste moisture, aeration, fragmentation, etc., are still unknown. Moreover, studies focused on identification of the bacteria responsible for CO production during composting has not been conducted and the link between the composting process and the activity of CODH enzyme, which may be the crucial element of this issue, is still unknown.

Due to the gaps in the literature, the current studies of CO emissions from the aerobic processes can lead to results that are burdened with high uncertainty. It is recommended to conduct comprehensive basic research on optimal parameters for CO production during bio-waste composting. Determining the impact of individual variables, such as aeration and temperature, will allow the development of a mathematical model to control and intensify CO production from this process. Further studies on the isolation and identification of the bacterial species, potentially responsible for the CO production are needed. It is also necessary to analyze the expression of CODH encoding gene at different composting conditions.
